# Dopant-Free Hole Transporting Material Based on Nonconjugated Adamantane for High-Performance Perovskite Solar Cells

**DOI:** 10.3389/fchem.2021.746365

**Published:** 2021-10-25

**Authors:** Dongyu Fan, Ren Zhang, Yuheng Li, Chengwei Shan, Wenhui Li, Yunhao Wang, Feiyang Xu, Hua Fan, Zonghao Sun, Xuehui Li, Mengshuai Zhao, Aung Ko Ko Kyaw, Gongqiang Li, Jianpu Wang, Wei Huang

**Affiliations:** ^1^ Key Laboratory of Flexible Electronic (KLOFE) & Institute of Advanced Materials (IAM), Jiangsu National Synergistic Innovation Center for Advanced Materials (SICAM), Nanjing Tech University (NanjingTech), Nanjing, China; ^2^ Guangdong University Key Laboratory for Advanced Quantum Dot Displays and Lighting, and Department of Electrical & Electronic Engineering Southern University of Science and Technology, Shenzhen, China; ^3^ Wuhan National Laboratory for Optoelectronics (WNLO), Huazhong University of Science and Technology (HUST), Wuhan, China; ^4^ Shaanxi Institute of Flexible Electronics (SIFE), Northwestern Polytechnical University (NPU), Xi’an, China

**Keywords:** dopant free, hole transporting material, adamantane, perovskite solar cells, inverted planar structure

## Abstract

A new dopant-free hole transporting material (HTM) 4′,4‴,4‴'',4‴''''-(adamantane-1,3,5,7-tetrayl)tetrakis(N,N-bis(4-methoxyphenyl)-[1,1′-biphenyl]-4-amine) (Ad-Ph-OMeTAD) (named FDY for short), which consists of a nonconjugated 3D bulky caged adamantane (Ad) as the core, triphenyl amines as side arms, and phenyl units as a linking bridge, is synthesized and applied in an inverted planar perovskite solar cell (PSC). As a result, the champion device with FDY as HTM yields an impressive power of conversion efficiency (PCE) of 18.69%, with J_SC_ = 22.42 mA cm^−2^, V_OC_ = 1.05 V, and FF = 79.31% under standard AM 1.5G illumination, which is ca. 20% higher than that of the device based on PEDOT:PSS (only 15.41%). Notably, the stability of PSC based on FDY is much better than that of devices based on PEDOT:PSS, and the corresponding devices retain over 90% of their initial PCEs after storing for 60 days in a nitrogen glove box without any encapsulation. Even when stored in an open air condition with 50–60% relative humidity for 188 h, the retained PCE is still over 81% of its initial one. All these results demonstrate that the new design strategy by combing the bulky and nonconjugated (aliphatic) adamantane unit as the core and triphenyl amines as side arms can efficiently develop highly efficient HTMs for PSCs, which is different from the traditional way based on conjugated backbones, and it may open a new way for scientists to design small-molecule HTMs for PSCs.

## Introduction

As one of the new-generation photovoltaic technologies, perovskite solar cells (PSCs) herald the arrival of the new energy era because of their rapidly great progresses during the last two decades (Burschka et al., 2013; Lee et al., 2012; Bi et al., 2013; Jeon et al., 2015). The best recorded power conversion efficiency (PCE) of PSCs is up to 25.5% with a regular mesoporous structure and over 23% with an inverted planar (p-i-n type) structure (Li et al., 2020), due to the unique optoelectronic properties of perovskite materials (Stranks et al., 2013; Guo et al., 2018; Li et al., 2020). As one of the key components in high-performance PSCs, hole transporting materials (HTMs) (Xing et al., 2013) play several key roles such as the following: 1) the hole extraction and transferring; 2) reducing energy loss between the perovskite layer and anode; 3) affecting crystallization and morphology of perovskite in inverted devices; and 4) improving stability of PSCs by preventing infusion of O_2_ and moisture into the perovskite layer in regular devices. However, very few HTMs can satisfy the requirements of PSCs so far, and even the widely used HTMs such as poly (3,4-ethylenedioxythiophene)/poly (styrene sulfonic acid) (PEDOT:PSS), polytriarylamine (PTAA), and doped Spiro-OMeTAD still have many drawbacks. For PEDOT:PSS, 1) the mismatch between its work function and the HOMO energy level of perovskite usually yields low open-circuit voltage (*V*_*OC*_) of the device; (Meng et al., 2016) and 2) the hydrophilicity and acidity of PEDOT:PSS can result in a degradation of perovskite, leading to low stability of PSCs, while for PTAA, 1) its grave hydrophobicity usually causes a poor interfacial contact between PTAA and the perovskite layer, resulting in the charge recombination loss further (Huang et al., 2015); 2) the necessary dopants, such as F4-TCNQ, to improve the mobility of PTAA may increase a tendency of ion migration and decrease the long-term stability of device (Zhou et al., 2018); and 3) the high cost of PTAA limits its application in large-scale manufacturing (Pham et al., 2017). Regarding Spiro-OMeTAD, the necessary additives such as Li-TFSI and 4-tert-butylpyridine (tBP) usually cause ion migration and degradation of perovskite, both of which make the PSCs unstable. So, developing highly efficient but cheap and easily synthesized dopant-free HTMs is urgent but challenging.

During the last two decades, lots of efforts have been carried out to find highly efficient HTMs for PSCs and many inorganic HTMs (Liu et al., 2018a; Yang et al., 2018; Yu and Sun, 2018) (such as NiOx and CuI), organic HTMs including polymers (Sun et al., 2018; Jung et al., 2019) (such as P3HT, PTAA, PPY2, and PPE1), and organic small molecules (Liu et al., 2018b; Wang et al., 2019) (such as MPA-BTTI and DFH) have been investigated and achieved a great progress with PCEs over 20%.

**SCHEME 1:**
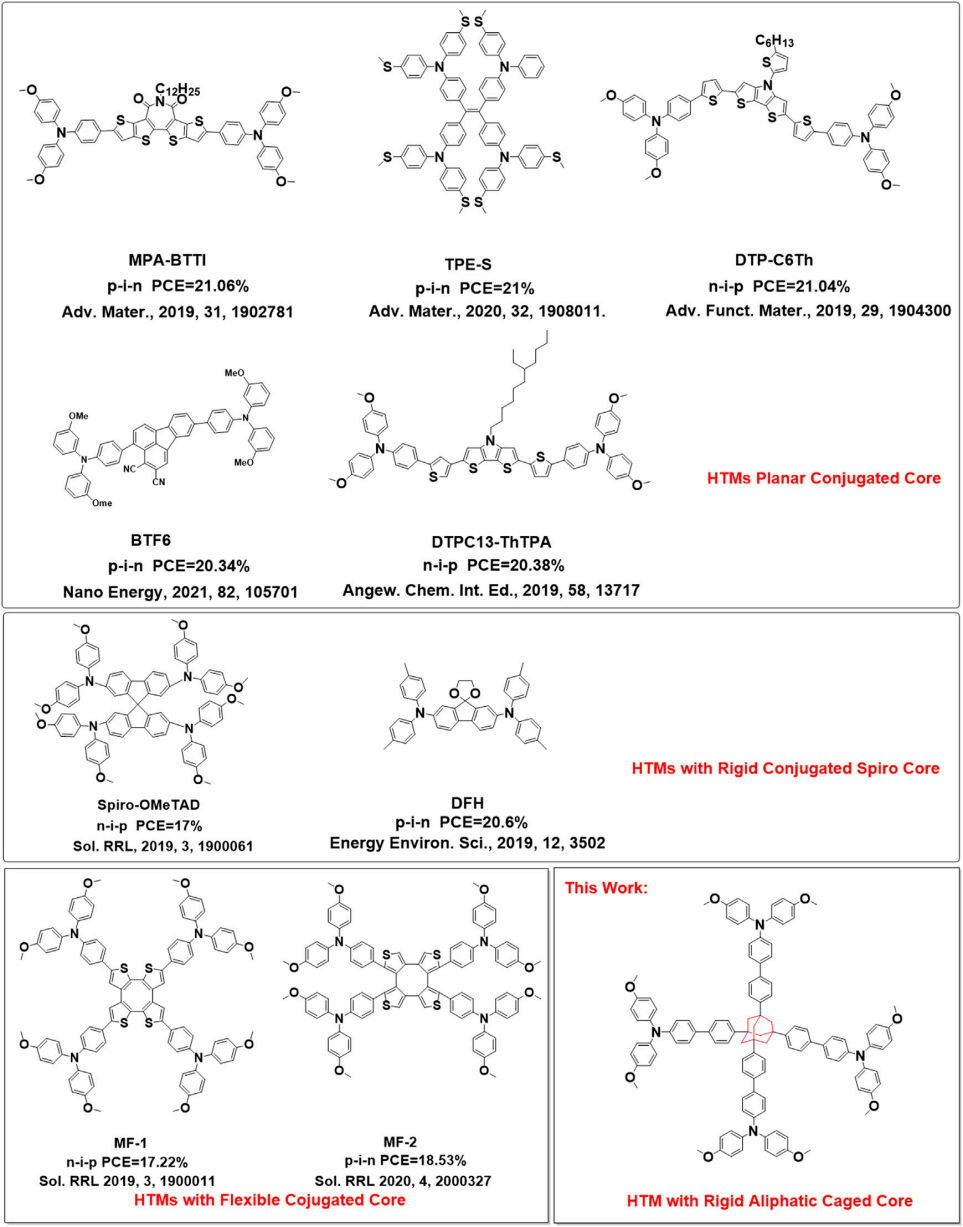
Strategies of HTMs Based-on Small Molecules.

Compared with inorganic HTMs and polymer HTMs, organic small molecule–based HTMs (SM-HTMs) have many advantages, such as the following: 1) tunable HOMO/LUMO energy levels; 2) easy synthesis and purification; and 3) very good batch-to-batch repeatability. So, more and more scientists worked on SM-HTMs during the last decade. Scheme 1 summarizes most of the dopant-free SM-HTMs with excellent PCEs for PSCs (Saliba et al., 2016). After analyzing their chemical structures, it is obviously found that most of the HTMs are constructed with π-conjugated backbones to keep their good intermolecular π–π stacking in order to yield high mobility (Chen et al., 2019; Pham et al., 2019; Cai et al., 2019; Yin et al., 2020). However, some of them such as Spir-OMeTAD (Jiang et al., 2019a; Jiang et al., 2019b) and DFH (Cao et al., 2019) (shown in Scheme 1) are constructed with a rigid spiro core unit, which usually destroys the intermolecular π–π stacking and decreases the hole mobility. The nonconjugated and rigid spiro structures demonstrate that the conjugated backbones in HTMs may not be necessary, and this inspires us to explore a new way to design HTMs for high-performance PSCs. In 2019, we first designed saddle-shaped HTMs named α, β-COTh-Ph-OMeTAD (Lai et al., 2019a) with the strategy of flexible core with tunable conformation (FCTC), in which the configuration of α, β-cycloocta[1,2-b:4,3-b′:5,6-b′:8,7-b′′′]tetrathiophenyl (α, β-COTh) (Rezaee et al., 2018) can be tuned based on the interactions between side arms and perovskite or interactions between side arms themselves, which may lead to a good balance of mobility and charge recombination, and achieved the champion PCE of 17.22% under AM 1.5 conditions with the regular mesoporous device structure. Later on, Nazaruding’s group (Snaith and Grätzel, 2006) and Yang’s group (van Duren et al., 2004) also independently reported similar results with the PCE of 16.3 and 17.7%, respectively. In 2020, we synthesized β, β-COTh-Ph-OMeTAD (Meng et al., 2020) and achieved a PCE of 18.53% in inverted planar PSCs. Recently, Wang’s group (Wang et al., 2021) and Ahmad’s group (Urieta-Mora et al., 2019) extended the COTh to COPh (o-tetraphenylene) and COI (tetra-indole) and synthesized OTP-OMeDPA and TTI, achieving a PCE of 21.5 and 19.23% in regular mesoporous PSCs, respectively. All these results demonstrate that even when the conjugate of backbones are partially destroyed with a flexible 3D structure of cyclooctatetraene in HTMs, they still can lead to high-performance PSCs. So, how is the conjugated core important in HTMs? The curiosity inspired us to design a unique structure of HTMs with adamantane (Ad) as the core and triphenylamines as side arms, in which the Ad structure is an aliphatic cage unit and can destroy the conjugation of HTM completely. To reduce the negative influence of the bulky structure of Ad on the intermolecular π–π stacking of side arms and enhance hole mobility of new HTM, we introduced a phenyl group as a bridge between Ad and side arms. The structure of new HTM (Ad-Ph-OMeTAD) (named FDY for short) is shown in **Scheme 1**, and its highest occupied molecular orbital (HOMO)/lowest unoccupied molecular orbital (LUMO) energy levels are −5.24/−2.09 eV, respectively. We fabricated the inverted planar PSCs with a structure of ITO/ HTM/ perovskite / PC_61_BM/ BCP/ Ag in which either PEDOT:PSS or FDY was as a dopant-free HTM. As a result, the devices based on FDY have a champion PCE of 18.69% with a J_SC_ of 22.42 mA cm^−2^, V_OC_ of 1.05 V, and FF of 79.31% under the standard AM 1.5G illumination, which is around 20% higher than that of devices based on PEDOT:PSS (PCE = 15.41%, J_SC_ = 19.38 mA cm^−2^, V_OC_ = 1.02 V, and FF = 78.08%). These results demonstrate that hole transport materials with a rigid nonconjugated structures such as adamantane as the core also have great potential for high-performance PSCs, and the new strategy may open a new way to design hole transport materials for high performance of PSCs.

**GRAPHICAL ABSTRACT:**
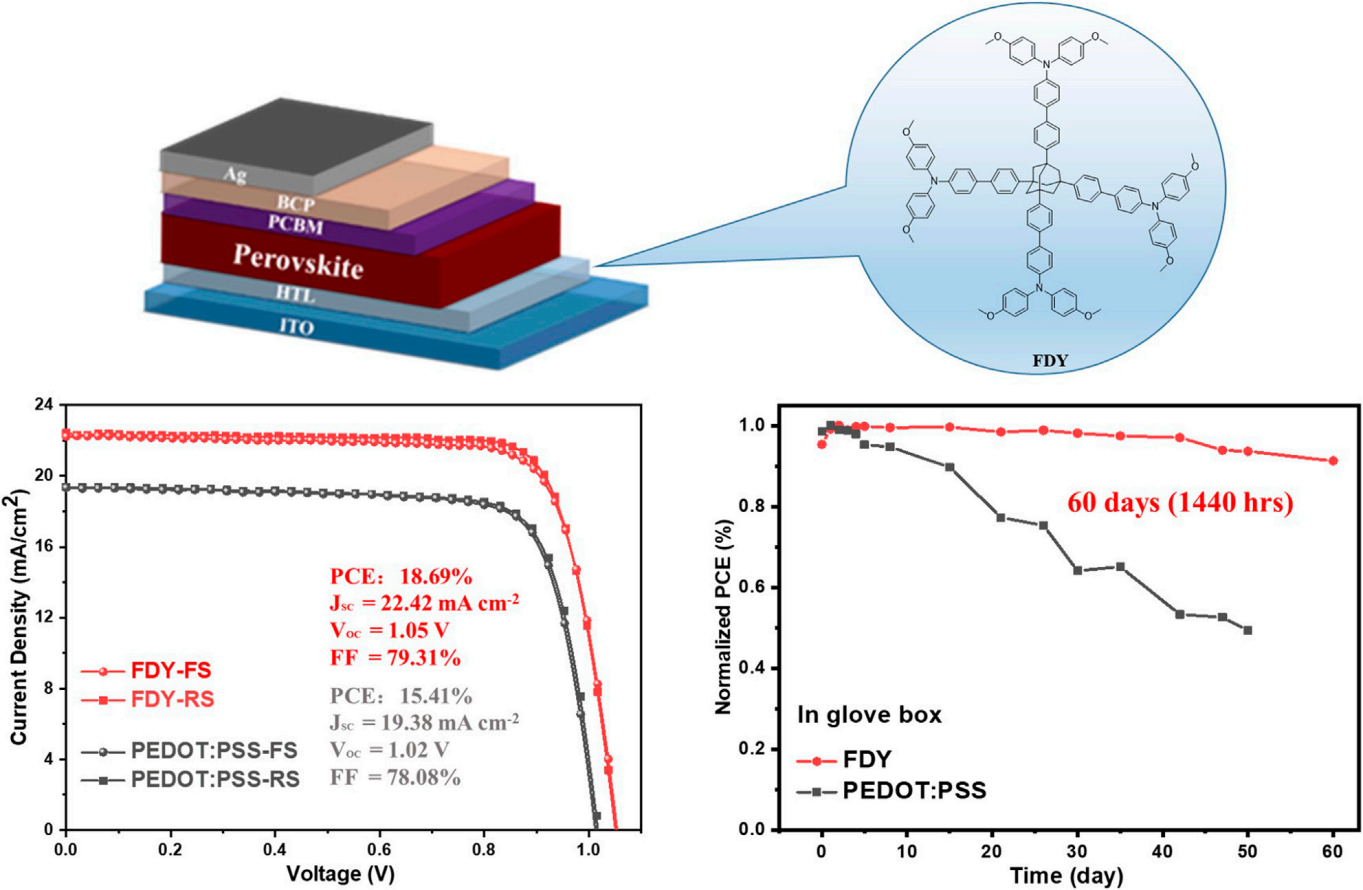
A new hole transporting material (HTM) Ad-Ph-OMeTAD (named FDY for short) with a nonconjugated 3D bulky caged adamantane (Ad) as the core is reported and applied in an inverted planar perovskite solar cell (PSC) with a power of conversion efficiency of 18.69%.

## Results and Discussion

### Synthesis and Properties of HTM (Ad-Ph-OMeTAD)

As shown in Scheme S1, the compound FDY was synthesized from commercially available compounds 1,3,5,7-tetrakis(4-iodophenyl) adamantane and (N, N-bis(4-methoxyphenyl)-4-(4,4,5,5-tetramethyl-1,3,2-dioxaborolan-2-yl)) *via* Suzuki cross-coupling with good yield. The structure was characterized by nuclear magnetic resonance (NMR) spectroscopy (Supplementary Figures S1–2, Supporting Information). The thermal properties were determined by thermogravimetric analysis and differential scanning calorimetry, respectively. The compound FDY exhibits an appropriate thermal stability with a phase transition temperature at 91°C and a decomposed temperature at 400°C (Supplementary Figures S4A,B, Supporting Information). Due to the bulky three-dimensional structure of adamantane, the intermolecular interaction is weak and the glass transition temperature is low. This maybe a benefit to form a better stacking mode of side arms when the film of FDY is treated with thermal annealing and enhance the hole extraction and the transport efficiency of FDY films.

As shown in Supplementary Figure S3A, the UV–vis absorption of FDY film is mainly at 300–400 nm, and the absorption positions of the solution and the film are similar, indicating that the intermolecular π–π stacking of FDY in the film is relatively weak. According to the onsite of the UV–vis absorption spectrum, we calculated the optical band gap of FDY is 3.15 eV, and the HOMO/ LUMO energy levels of FDY were determined by ultraviolet photoelectron spectroscopy (UPS) measurement, as shown in Supplementary Figure S3. The valence regions (*E*_onset_) and secondary electron cutoff edges (*E*_cutoff_) are determined to be −8.94 eV (Supplementary Figure S3D) and 7.04 eV (Supplementary Figure S3E), respectively. The HOMO/LUMO energy levels of FDY are calculated to be −5.24 eV and −2.09 eV, respectively. Compared with PEDOT:PSS (−5.0 eV) and PTAA (−5.22 eV), the HOMO energy level of FDY matches much better with the valence band maximum of MAPbI_3_; meanwhile, the lower LUMO energy level of FDY can effectively block the migration of electrons and avoid a nonradiative recombination at the interface between perovskite and HTM, which usually may lead to a higher Voc.

The mobility of FDY was determined by the space charge limited current (SCLC) method with a hole-only device based on a structure of ITO/PEDOT: PSS/FDY/Au, and it is determined to be 7.62 × 10^–5^ cm^2^ V^−1^s^−1^ (Supplementary Figure S5, Supporting Information) (Snaith and Grätzel, 2006). All optical and electrochemical data of FDY are summarized in Table 1.

**TABLE 1:** Optical and electrochemical properties of FDY.

	*λ*_sol_ [nm]^a^	*λ*_film_ [nm]^b^	*E*_g_^opt^ [eV]^c^	*E*_HOMO_ [eV]^d^	*E*_LUMO_ [eV]^e^	*μ*_h_ [cm^2^ V^−1 s−1^]^f^
FDY	384	394	3.15	−5.24	−2.09	7.62 × 10^−5^

a Solution absorption spectra (2 mg/ml in toluene).

b Absorption spectra of films on quartz glass.

c Optical band gap (*E*_g_^opt^) estimated from the edge of absorption spectra.

d Determined by ultraviolet photoelectron spectroscopy (UPS) measurement.

e *E*_LUMO_ = *E*_HOMO_ + *E*_g_^opt^.

f Measured by the SCLC method.

### Device Performance and Characterization

**FIGURE 1:**
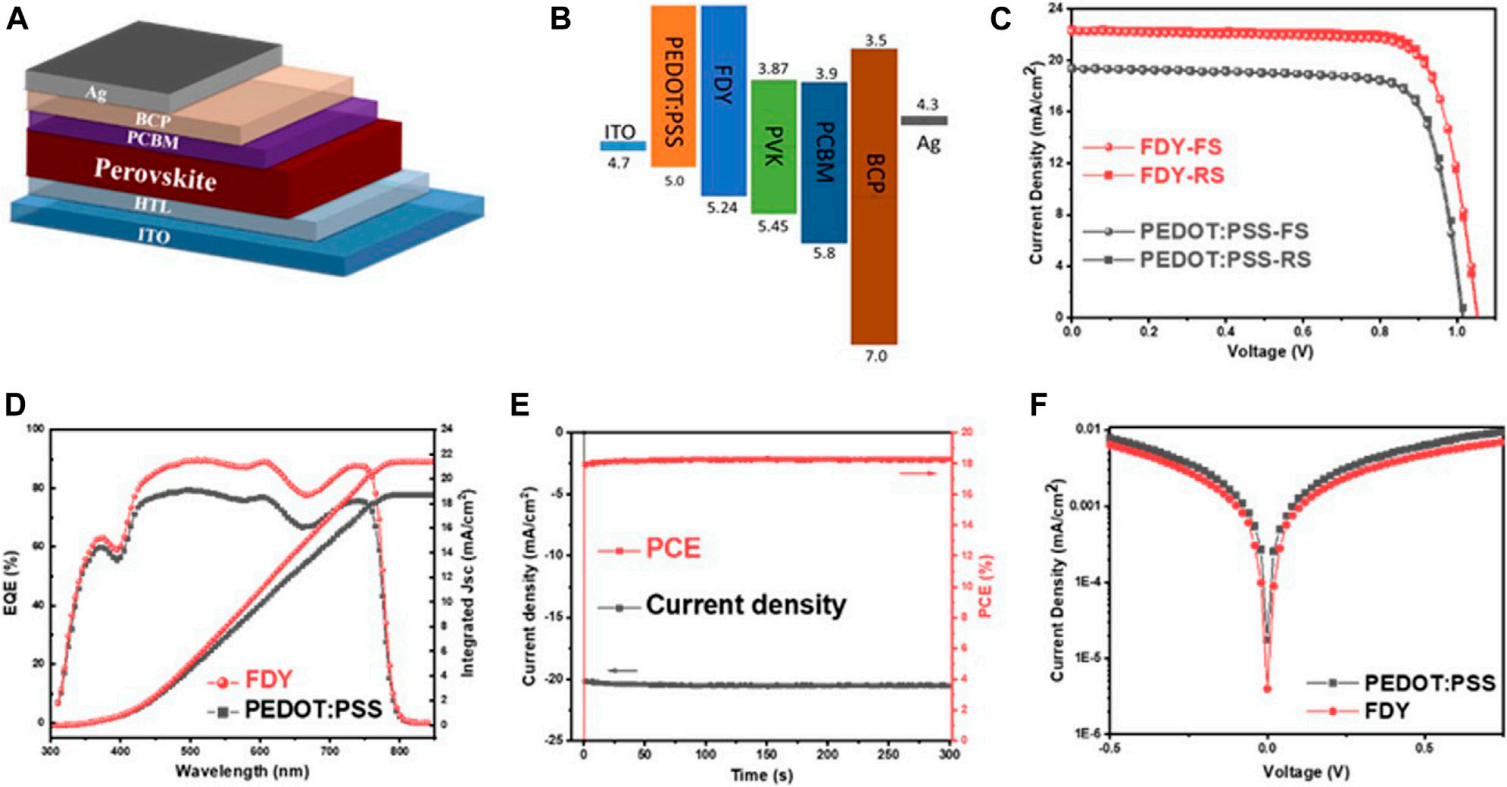
**(A)** Device structure and **(B)** energy levels of individual layers used in the device, comparing the energy levels of FDY and PEDOT:PSS. **(C)** *J–V* curves (forward and reverse scans) of the best PSCs prepared by PEDOT:PSS and FDY HTL under AM1.5G illumination, showing negligible hysteresis. **(D)** EQE spectrum and corresponding integrated short-circuit current density. **(E)** The steady-state efficiency of the champion device based on FDY HTL at the maximum power point for 300 s. **(F)** The dark *J–V* curves of the devices.

To evaluate the performance of FDY as dopant-free HTM in inverted perovskite solar cells, we fabricated the corresponding devices with a structure of ITO/ FDY/ MAPbI_3_/ PC_61_BM/ BCP/ Ag and the control devices with PEDOT:PSS as HTM for comparison (Figure 1A), and the energy levels of respective layers are shown in Figure 1B. For all devices, the light-absorbing layer was prepared with MAPbI_3_ by the one-step antisolvent method. The concentrations of FDY were optimized to improve the performance of PSCs (Supplementary Table S1), and the best PCE of 18.69% with a J_SC_ of 22.42 mA cm^−2^, V_OC_ of 1.05 V, and FF of 79.31% was achieved with a concentration of 2.0 mg ml^−1^ in toluene and the hysteresis was negligible, which is much better than that of the control device based on PEDOT:PSS (PCE = 15.41% with a J_SC_ of 19.38 mA cm^−2^, V_OC_ of 1.02 V, and FF of 78.08%). Figure 1C displays the current density–voltage (*J*–*V*) curves for the optimized devices under the standard AM 1.5G illumination, and the results are summarized in Table 2. The EQE spectrum was executed to confirm J_SC_ values obtained from the *J*–*V* curve tested under the solar simulation, as shown in Figure 1D. The integrated J_SC_ from EQE is calculated to be 21.52 mA cm^−2^ for FDY and 18.71 mA cm^−2^ for PEDOT:PSS-based devices, respectively. Both match very well with the current from the *J–V* curve. Furthermore, we measured the stabilized power outputs for 300 s at the maximum power point. The PCEs obtained from the stabilized power outputs are 18.31 and 14.61% for the devices based on FDY and PEDOT:PSS (Figure 1E), respectively, indicating a good reliability of the devices. As shown in Figure 1F, the dark current of the devices with FDY and PEDOT:PSS was measured, and the device with FDY in a thickness of 10 nm (2 mg ml^−1^) showed a lower leakage current, implying there was less recombination in the device.

**TABLE 2:** Performances of PSCs based on PEDOT:PSS and FDY.

HTL	Scanning	*J*_sc_ [mA cm^−2^]	*V*_oc_ [V]	FF [%]	Best PCE [%]
PEDOT:PSS	Forward	19.34	1.01	77.87	15.26
	Reverse	19.38	1.02	78.08	15.41
FDY	Forward	22.22	1.05	77.96	18.26
	Reverse	22.42	1.05	79.31	18.69

**FIGURE 2:**
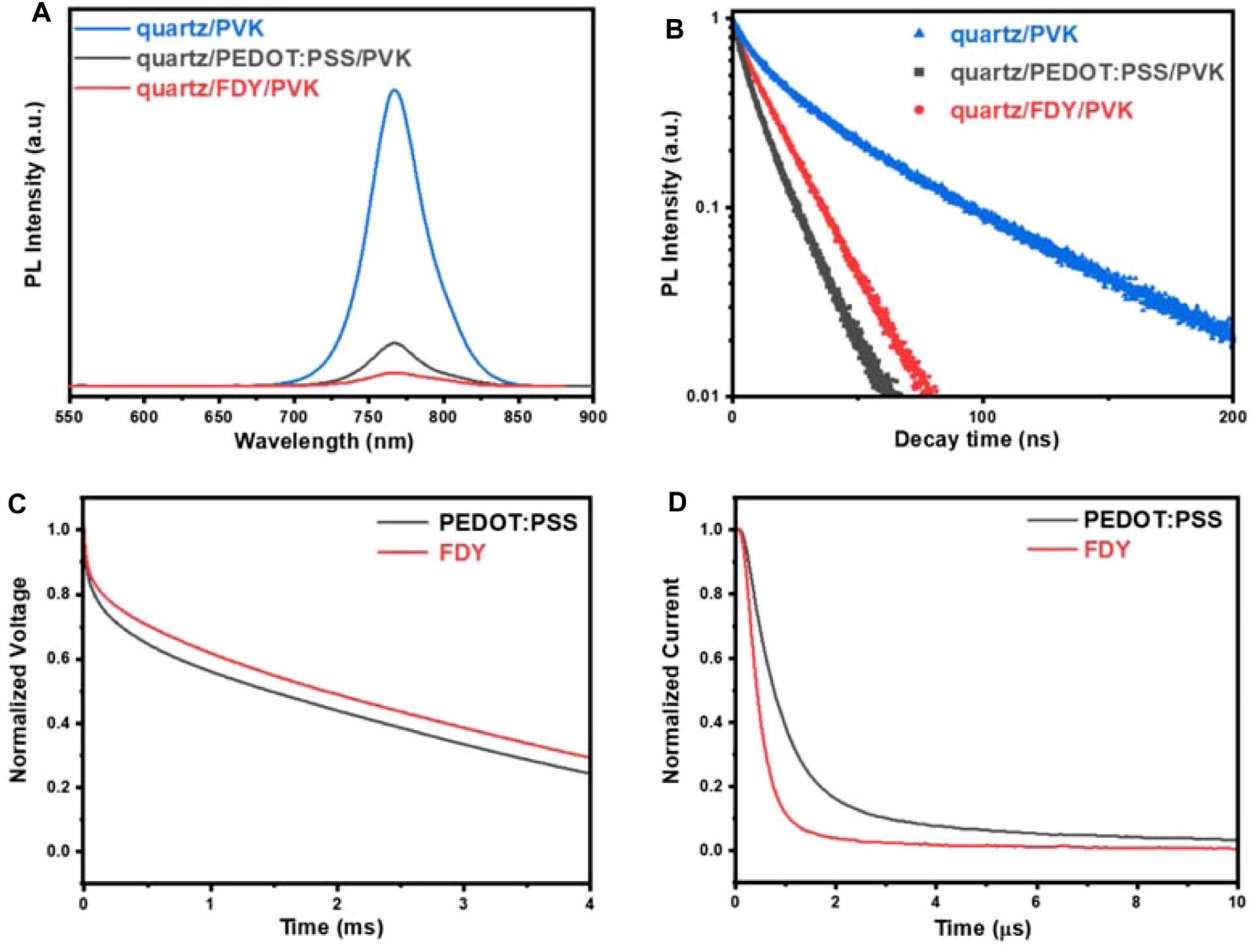
**(A)** Steady-state PL spectra and **(B)** the decay curves of the TRPL intensity of the bare perovskite film and those fabricated on FDY and PEDOT:PSS upon illumination with a 450 nm laser. **(C)** Transient photovoltage characteristics and **(D)** transient photocurrent characteristics of devices based on PEDOT:PSS and FDY HTL.

To understand the role of FDY on PSCs further, steady-state photoluminescence (PL) and time-resolved photoluminescence (TRPL) were measured. From Figure 2A, it is obviously found that the peak intensity of PL of perovskite on FDY (with a quench efficiency of 95.5%) was weaker than that on PEDOT:PSS (85.4%), indicating that the FDY as HTM can extract holes from the perovskite layer much easier than PEDOT:PSS. As literature reported, HTM as the substrate in inverted planar devices usually affects the quality of the perovskite film (Lai et al., 2019b). From the TRPL tests (Figure 2B), we obtained the carrier lifetimes of perovskite films based on FDY and PEDOT:PSS, and the details are summarized in Supplementary Table S2. The photocarrier lifetimes of perovskite films depositing on FDY are τ_1_ = 2.86 ns and τ_2_ = 25.28 ns, and those of PEDOT:PSS are τ _1_ = 8.11 ns and τ_2_ = 14.48 ns. Shorter τ_1_ lifetime means that the FDY film has stronger hole extraction ability and the hole can be transferred from perovskite to HTL more quickly. The results of τ_2_ indicate that the perovskite films prepared on FDY films have fewer defects and excitons can exist for a longer time in perovskite films before recombination.

Transient photovoltage (TPV) and transient photocurrent (TPC) tests were also carried out, which are often used to characterize carrier lifetimes and carrier extraction efficiency in organic solar cells (Clarke et al., 2014). The longer lifetime of TPV indicates the longer lifetime of generated carriers in an open-circuit state, demonstrating the lesser nonradiative recombination in the device. The TPV measurement in Figure 2C shows that the carrier lifetime of the device on FDY is significantly longer than that on the PEDOT: PSS. It means that the nonradiative recombination at the interface of FDY and perovskite is less than that of PEDOT:PSS, which is consistent with the higher V_OC_ of devices based on FDY HTL. Meanwhile, as shown in Figure 2D in the TPC test, the lifetime represents the stay time of the photogenerated carriers in the short-circuit state of the device, and the device on FDY has a TPC decay faster than that on PEDOT:PSS, indicating that FDY has a stronger hole extraction ability and faster hole transfer ability from the perovskite layer to the electrode. The longer decay TPV and shorter decay TPC of the device on FDY indicate that the FDY HTL improves the extraction efficiency of holes while reducing the nonradiative recombination in the interface, which may lead to a higher Voc than PEDOT:PSS.

To further investigate the charge recombination of the devices based on FDY, the light intensity dependence of *J–V* characteristics was conducted. The slope of V_OC_ versus natural logarithm of illumination intensity gives *kT*/*q* in a trap-free system, where *k*, *T*, and *q* are the Boltzmann constant, temperature in Kelvin, and the elementary charge, respectively (Cao et al., 2019). As shown in Supplementary Figure S6, the fitting results for *n* of PEDOT:PSS and FDY are 1.51 and 1.36, respectively. A higher value of *n* indicates that PEDOT: PSS causes a serious monomolecular recombination in the device when used as HTL; in contrast, FDY can reduce the trap density of the device and inhibit charge recombination when used as HTL, which corresponds to the PL and TRPL measurements.

**FIGURE 3:**
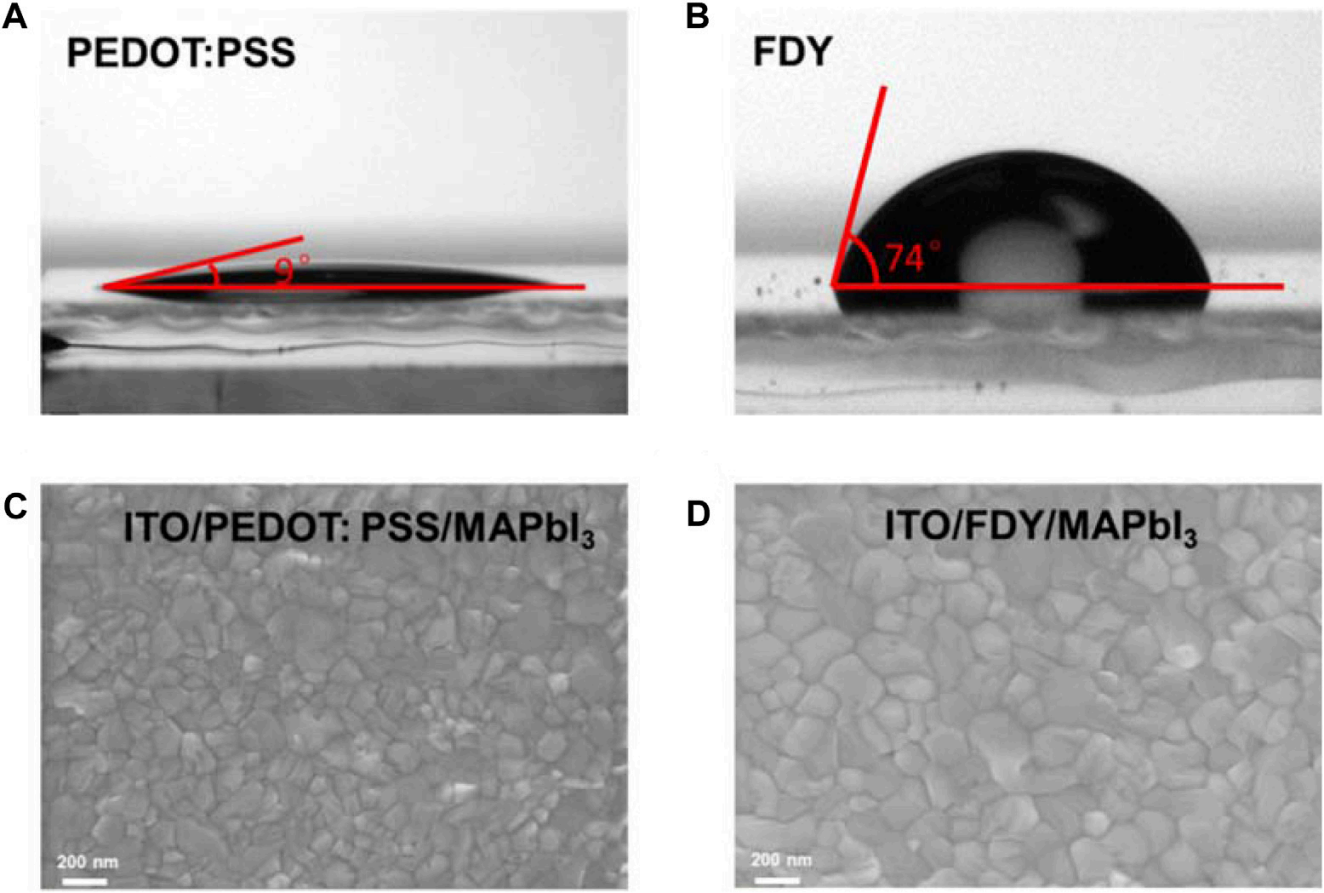
The contact angle images of **(A)** PEDOT:PSS and **(B)** FDY films; top surface SEM of perovskite films fabricated on **(C)** PEDOT:PSS and **(D)** FDY HTL.

In order to compare the different effects of FDY and PEDOT:PSS on the perovskite layer, AFM was used to characterize the roughness of the HTM layer. As shown in Supplementary Figure S7 (Supporting Information), the root mean square roughness of PEDOT:PSS is 2.70 nm, while that of FDY is 2.88 nm, and both films are very smooth with almost the same roughness. However, the tests of contact angle demonstrate that the surface property of two films are very different, which affects the morphology of the perovskite layer noticeably. As shown in Figures 3A,B, the contact angle of PEDOT:PSS is only 9° with water, due to its high hydrophilicity, while that of FDY is 74°, which is much more hydrophobic and can effectively prevent the water from eroding the perovskite film and can also keep a suitable wettability with the perovskite precursor solution. All these will be conducive to the preparation of a homogeneous and dense perovskite film and enhancing the stability of devices (as discussed in the following).

The morphology of perovskite films on FDY and PEDOT:PSS was tested by Scanning Electron Microscopy (SEM) (Figures 3C,D). Compared with the film on PEDOT:PSS, the MAPbI_3_ perovskite film on FDY has much larger grain sizes and less grain boundaries, which leads to higher crystal quality of the perovskite layer and improves the charge extraction from perovskite to HTM. The results agree very well with that of PL, TRPL, TPV, and TPC.

**FIGURE 4:**
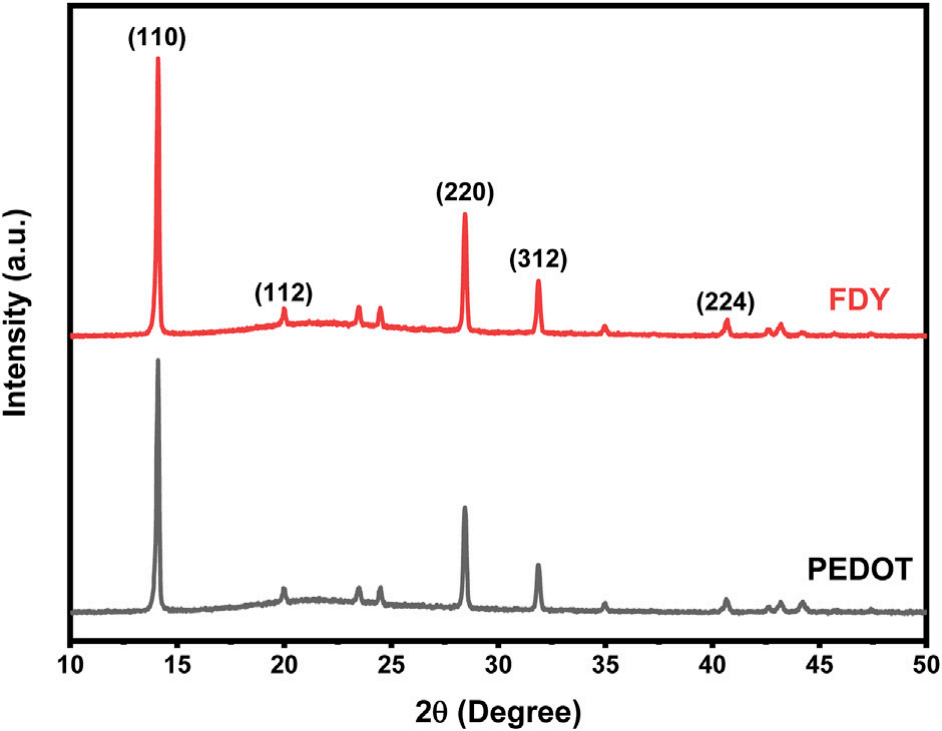
XRD of quartz /PEDOT: PSS/perovskite and quartz /FDY/ perovskite.

In addition, X-ray diffraction (XRD) characterization also shows that the perovskite films deposited on FDY have better crystallinity. As shown in Figure 4, the intensities of peaks of (110) and (220) of the perovskite film on FDY film are much higher than those on PEDOT:PSS comparing with the peak of (312). It shows that the hydrophobicity of the FDY film can not only increase the grain size of the perovskite film but also improve the crystallinity of the perovskite film.

**FIGURE 5:**
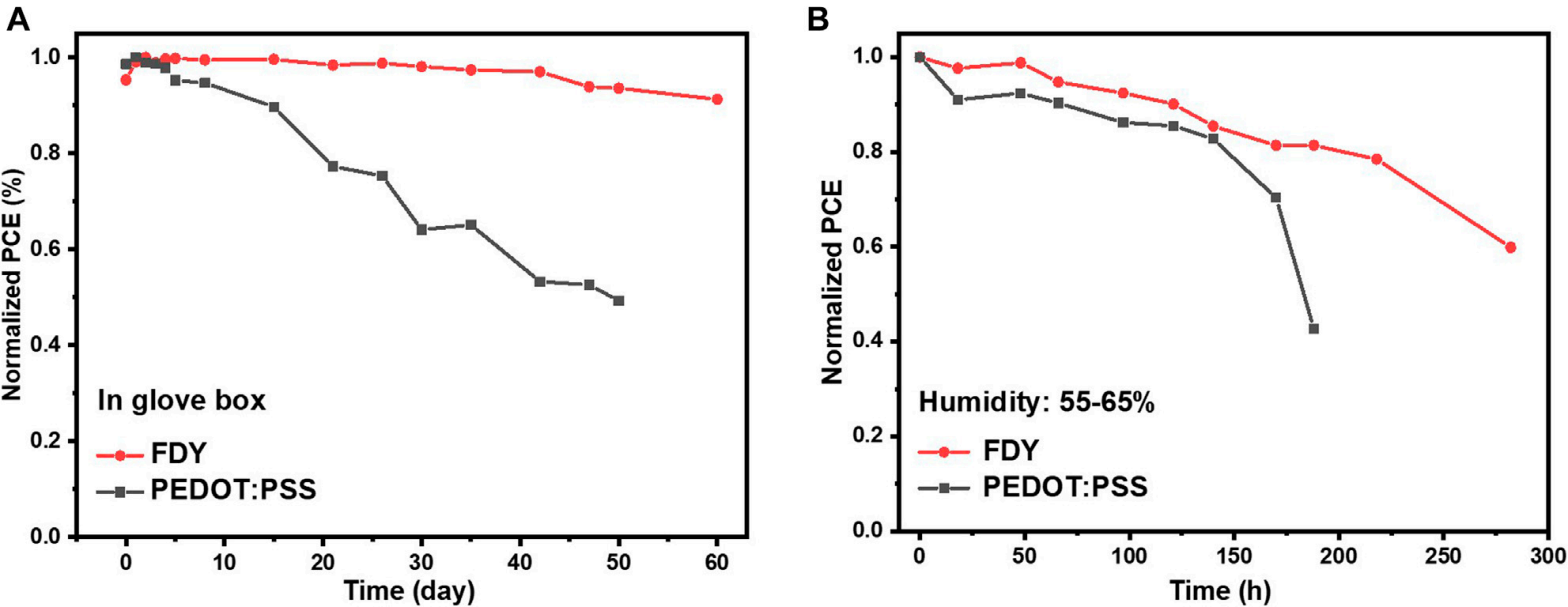
Stability test of the unencapsulated devices based on PEDOT:PSS and FDY HTL **(A)** stored in a N_2_ glove box in the dark at room temperature and **(B)** stored in air with 50–60% relative humidity.

As discussed above, the suitable hydrophobicity of FDY should be beneficial to the stability of PSCs not only by preventing the perovskite film from the moisture but also by improving the crystal quality of the perovskite film. The stabilities of PSCs based on FDY and PEDOT:PSS were measured as shown in Figure 5. Interestingly, the PCE of the device based on FDY was retained more than 90% of its initial one after storing for 60 days in a nitrogen glove box without any encapsulation while that of device based on PEDOT:PSS in the same condition dropped down to 50% in 50 days (Figure 5A), which may be caused by the corrosion of acidic PEDOT:PSS on the perovskite film. Moreover, when the devices based on FDY were stored for 188 h in an ambient air with 50–60% relative humidity (Figure 5B), the PCE still remained at 81% of its initial one, while that of the PEDOT:PSS-based device dropped down to 43% of its initial one. The excellent long-term stability of FDY-based devices may benefit from the high hydrophobicity of the FDY film and high quality of the perovskite film.

## Conclusion

In summary, we synthesize a new dopant-free SM-HTM with a nonconjugated aliphatic motif adamantane as the core and triphenyl amines as side arms and applied it in inverted planar PSCs. After systematical investigation, it is found that the HTM FDY can enhance the quality of perovskite films and decrease the nonradiative charge recombination and all of them can improve the PCE and stability of PSCs. The champion device with FDY yields an impressive PCE of 18.69% with Jsc = 22.42 mA cm^−2^, V_OC_ = 1.05 V, and FF = 79.31% under standard AM 1.5G illumination with a very impressive long-term stability, especially even stored for 188 h in an open air with 50–60% relative humidity without any encapsulation, the PCE still retained more than 80% of its initial one. Our results may open a new way to design SM-HTMs for high-performance PSCs by introducing an aliphatic nonconjugated bulky core such as adamantane and so on.

## Experiment

### Materials

Poly(3,4-ethylenedioxythiophene)/poly(styrene sulfonic acid) (PEDOT:PSS), PbI_2_ (99.99%, TCI), MAI (Aus), PC_61_BM (Xi’an P-OLED), 2,9-dimethyl-4,7-diphenyl-1,10-phenanthroline (BCP), (98%+, Aldrich), anhydrous N, N, dimethylformamide (DMF), anhydrous dimethyl sulfoxide (DMSO), toluene (99.8%), and anhydrous chlorobenzene (CB) were purchased from Sigma-Aldrich. All reagents were used directly without any further purification.

### Perovskite Precursors

650 mg PbI_2_ (99.99%, TCI) and 224.2 mg MAI (Aus) were dissolved in 1.0 ml mixed solvent of DMF:DMSO = 4:1. Then, the solution was stirred overnight at room temperature and then filtered with 0.22 µm syringe PTFE filters.

### Device Fabrication

PSCs with the structure of ITO/ PEDOT:PSS or FDY /MAPbI_3_/PC_61_BM/BCP/Ag were fabricated, wherein the ITO was the bottom layer. ITO glass substrates were sequentially washed with soap water, acetone, and isopropanol for 30 min and dried with N_2_. Then, the glasses were treated with a UV cleaner for 20 min. The PEDOT:PSS film was fabricated by spin-coating the PEDOT:PSS aqueous dispersion at 5,000 rpm for 45 s after filtering. Different concentrations of FDY were dissolved in toluene, and the films were spin-coated onto the ITO substrate at 5,000 rpm for 45 s and then were annealed at 150°C for 10 min. After being transferred into a glovebox, the perovskite films were spin-coated on substrates with a solution of 50 μl perovskite precursor at 4,000 rpm for 20 s and the antisolvent of 120 μl chlorobenzene was dropped on the perovskite layer during the 6th second. The films were annealed at 80°C for 10 min, immediately. An electron transport layer of PC_61_BM (99.5%, Xi’an P-OLED) was deposited from a chlorobenzene solution (20 mg ml^−1^) at 1,000 rpm for 40 s, and then, a bathocuproine (98% +, Aldrich) solution (0.5 mg ml^−1^ in ethanol) was spin-coated at 4,000 rpm for 40 s. Finally, device fabrication was completed by thermal evaporation of a 80 nm thick film with Ag as the cathode.

### Film Characterization

The 1H NMR spectra were measured on an MECUYR-VX300 spectrometer. The UV–vis absorption spectra were recorded on a Shimadzu UV-2500 recording spectrophotometer. Ultraviolet and UPS measurements were performed in an ultrahigh vacuum surface analysis system equipped with a SCIENTA R3000 spectrometer with a base pressure of 10^−10^ mbar. UPS employed the He I 21.22 eV as the excitation source with an energy resolution of 50 meV. XRD patterns were recorded using a PANalytical X-ray diffractometer with Cu Ka radiation for crystallinity comparison. The scanning step size was 0.01°.

### Device Characterization

The current–voltage (J-V) curves of the solar cells were characterized after fabrication without any preconditioning via a Keithley 2,400 system under an Enli Solar Simulator (AM 1.5 G,100 mW cm^−2^). The certified device active area of 0.11 cm^2^ was defined by the designed mask to avoid overestimation of the photocurrent density. NREL certified Si cells (KG-5) were used to calibrate the light intensity. The J–V curves were measured from 1.2 to 0 V (reverse scan) and 0–1.2 V (forward scan) with a step of 0.02 V in a glove box at RT. The EQE spectra were obtained using a QE-R system (Enli Tech.) under the AC mode. All of the PSCs had no encapsulation. Transient photovoltage decay was recorded by using a digital oscilloscope (DOS-X 3104A) at an open-circuit condition.

## Data Availability

The original contributions presented in the study are included in the article/Supplementary Material; further inquiries can be directed to the corresponding author.
