# Supplementary material for: Dopant-Free Hole Transporting Material Based on Nonconjugated Adamantane for High-Performance Perovskite Solar Cells
Source: Front Chem. 2021 Oct 25;9:746365. doi: 10.3389/fchem.2021.746365 (PMC8573366; doi:10.3389/fchem.2021.746365)
Supplement: Supplementary file 1 [file Image1.pdf]

Supporting Information

Dopant-Free Hole Transporting Material Based-on Non-conjugated Adamantane for High-Performance Perovskite Solar Cells

Dongyu Fan, Ren Zhang, Yuheng Li, Chengwei Shan, Wenhui, Li, Yunhao Wang, Feiyang Xu, Hua Fan, Zonghao Sun, Xuehui Li, Mengshuai Zhao, Aung Ko Ko Kyaw, Gongqiang Li*, Jianpu Wang, Wei Huang*

General information

All general reagents and chemicals were purchased from commercial sources (Aldrich, Acros, Strem, Matrix Scientific) and used without further purification. Reagent grade solvents were dried when necessary and purified by distillation. Experimental General Information ^{1}H NMR and ^{13}C NMR spectra were measured on a MECUYR-VX300 spectrometer. Elemental analyses of carbon, hydrogen, and nitrogen were performed on a Vario EL III microanalyzer. Mass spectra were measured on a ZAB 3F-HF mass spectrophotometer and Bruker autoflex matrix-assisted laser desorption/ionization time-of-flight (MALDI-TOF). UV-Vis absorption spectra were recorded on a Shimadzu UV-2500 recording spectrophotometer. Differential scanning calorimetry (DSC) was performed on a NETZSCH DSC 200 PC unit at a heating rate of 10 ^{o}C min-1 from -40 ^{o}C to 300 ^{o}C under argon. The glass transition temperature (Tg)

was determined from the second heating scan. Thermogravimetric analysis (TGA) was undertaken with a NETZSCH STA 449C instrument. The thermal stability of the samples under a nitrogen atmosphere was determined by measuring their weight loss while heating at a rate of 20 ºC min-1 from 25 to 500 ºC. Electrochemical spectra of **FDY** were measured by ultraviolet photoelectron spectroscopy (UPS) measurement system. The compounds **F2** (>98%)and **1** (95%) were purchased from Nanjing Ci-Llene Optoelectronics Science and Technology Co.,Ltd without any further purification .

Synthesis of Ad-Ph-OMeTAD

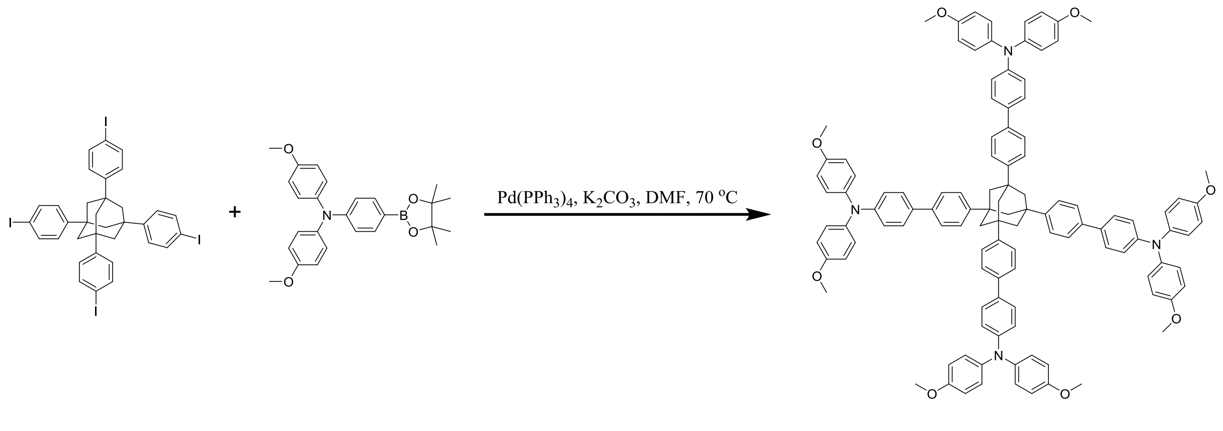

F2 **1** **FDY**

Scheme S1. Synthesis of **FDY**

Synthesis of **Ad-Ph-OMeTAD**:

Compound **F2** (480 mg, 0.5 mmol), compound **1** (976 mg, 2.26 mmol), $Pd(PPh_3)_4$ (115.6 mg, 0.1 mmol), K_2CO_3 (2 M, 2 mL) and DMF (50 mL) was placed in a Schlenk flask under an Nitrogen atmosphere and the mixture was stirred at 70 ℃ for 48 h.

After cooling down to room temperature and the mixture was extracted with DCM (50 mL) for three times. After combination, the organic layer was washed with brine for several times, dried over anhydrous $MgSO_4$. After removal of solvent, the residue was purified by chromatography with petroleum ether/CH_2Cl_2 (1:5, v:v) to get product Ad-Ph-OMeTAD (450 mg, 54%). ^{1}H NMR (400 MHz, Chloroform-d) δ 7.54 – 7.47 (m, 16H), 7.40 (d, J = 8.8 Hz, 8H), 7.08 (d, J = 9.0 Hz, 16H), 6.97 (d, J = 8.8 Hz, 8H), 6.83 (d, J = 9.0 Hz, 16H), 3.80 (s, 24H); ^{13}C NMR (100 MHz, Chloroform-d) 155.9, 148.4, 148.0, 141.1, 138.6, 133.0, 127.5, 126.7, 126.5, 125.5, 120.8, 114.8, 55.6, 48.3, 41.6, 38.3, 30.3. Elemental Analysis: C, 82.78; H, 6.09; N, 3.39; O, 7.74; C/H ratio 13.59. found: C, 82.40; H, 6.05; N, 3.43; O, 8.12; C/H ratio 13.61.

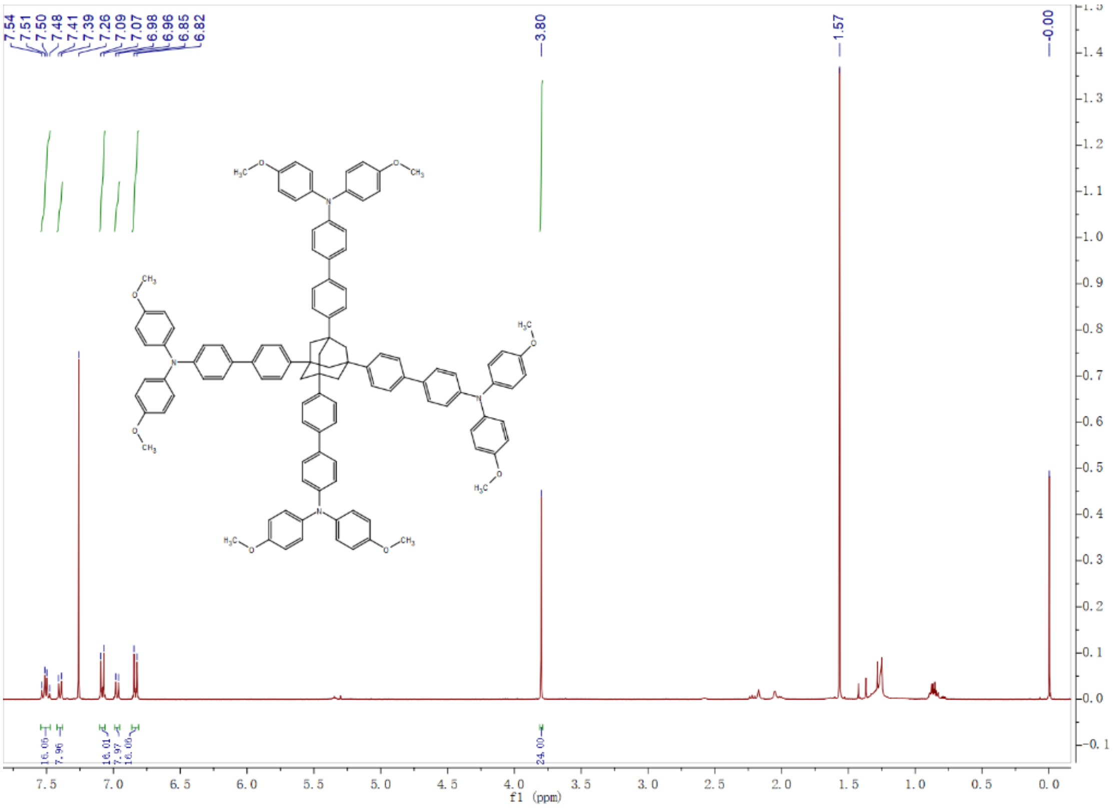

Figure S1 ^{1}H NMR spectrum of **FDY**

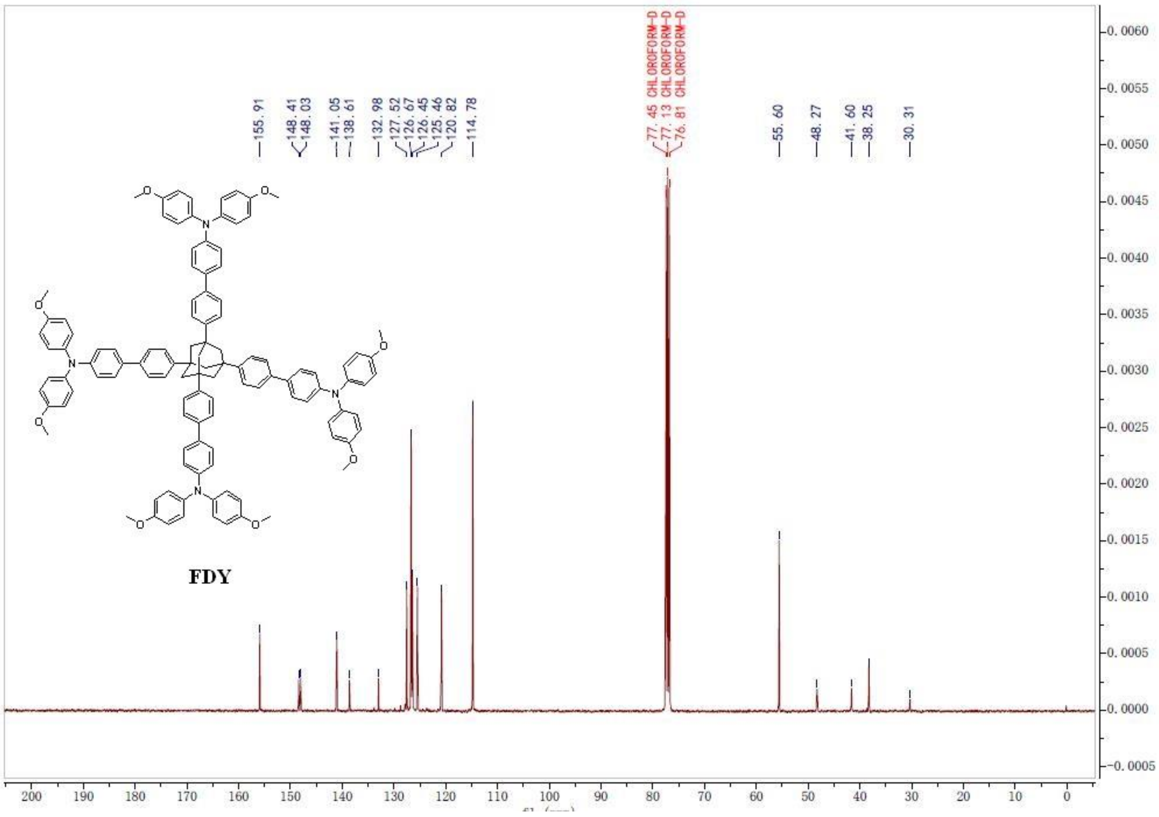

Figure S2 ^{13}C NMR spectrum of **FDY**

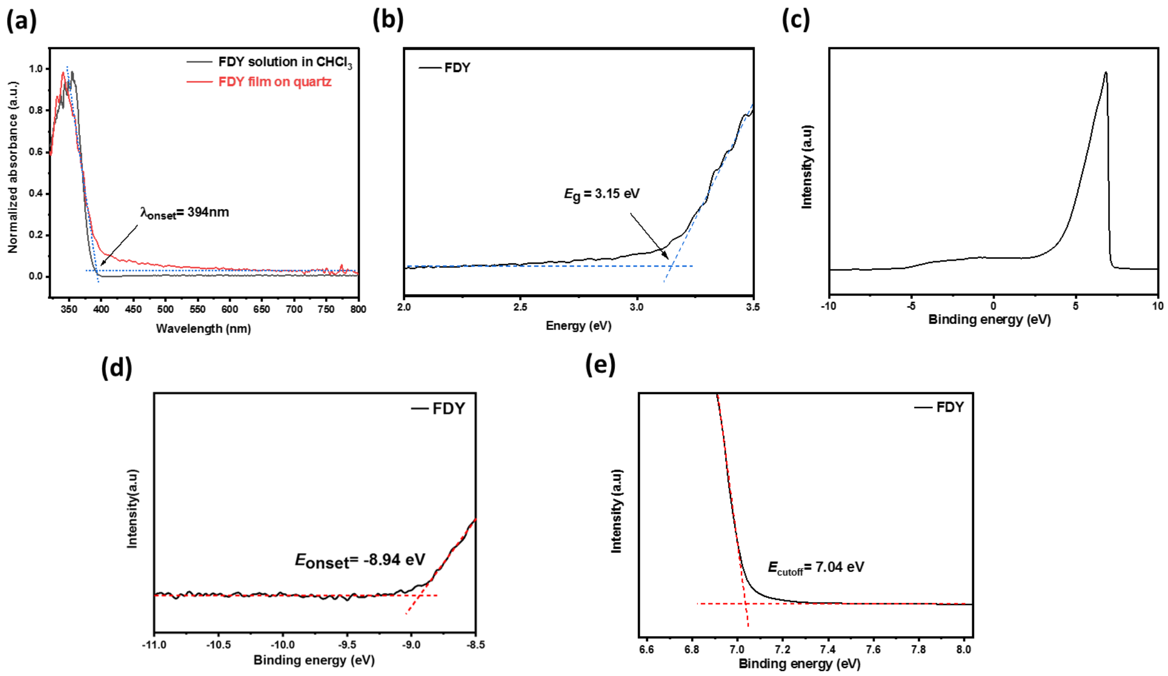

Figure S3. a) UV-Vis absorption spectrum of FDY film on quartz substrate, and solution in $CHCl_3$ (2.0 mg/mL); b) bandgap calculated from the edge of absorption spectrum. UPS spectra of the film of FDY: c) survey; d) valence regions; e) secondary electron cutoff

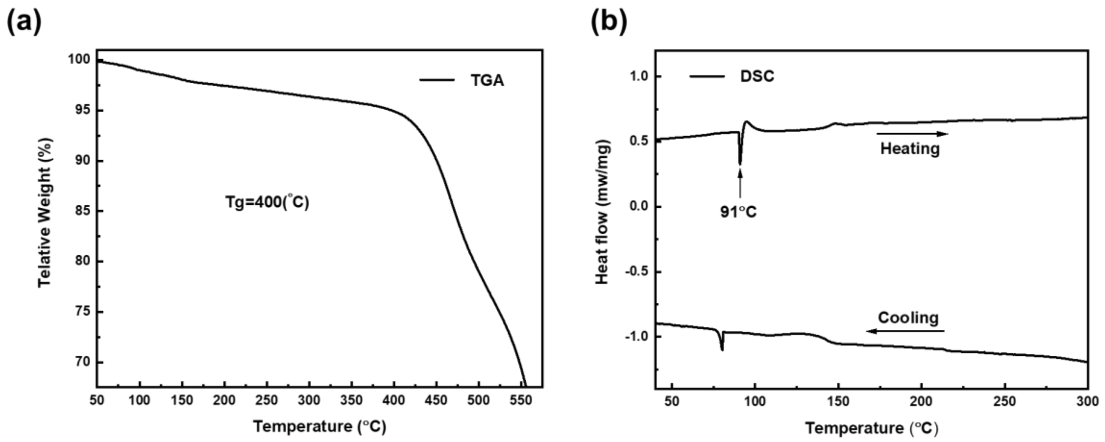

Figure S4. a) thermogravimetric analysis (TGA) curves, b) differential scanning calorimetry (DSC) trace of FDY measured under N_2 flow at heating and cooling rate of

10 °C/min (the second cycle).

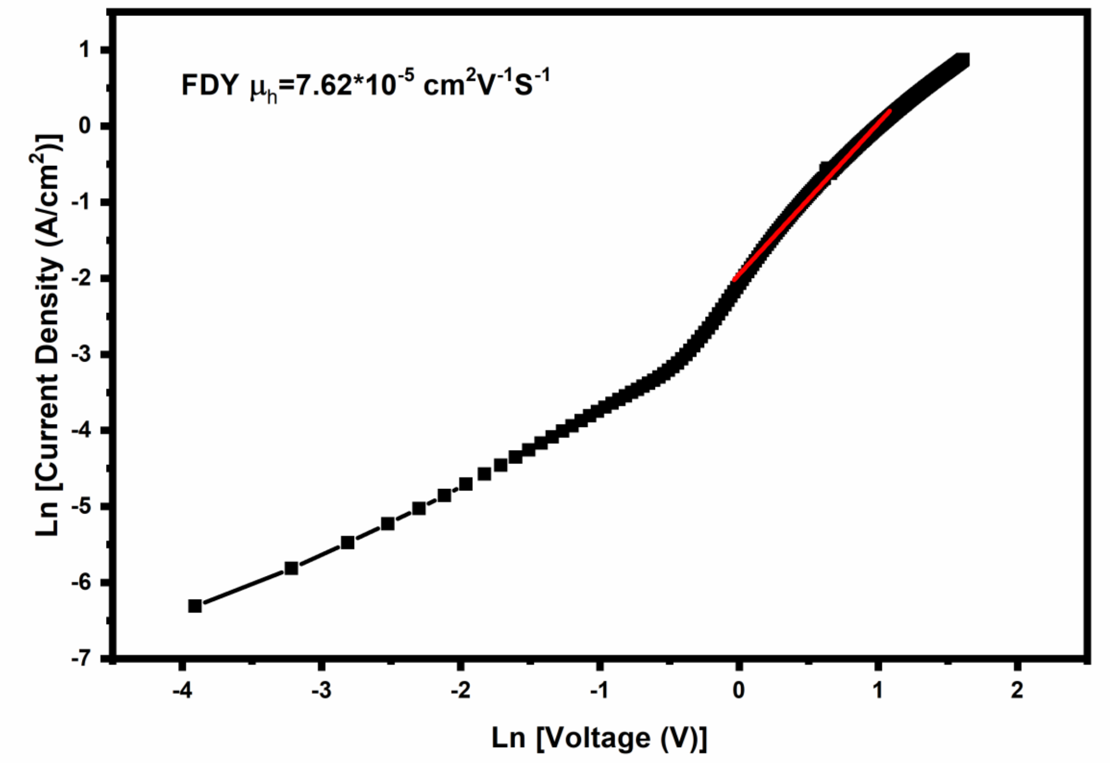

Figure S5. Mobility of FDY measured by SCLC and the film thickness of FDY is ~40 nm

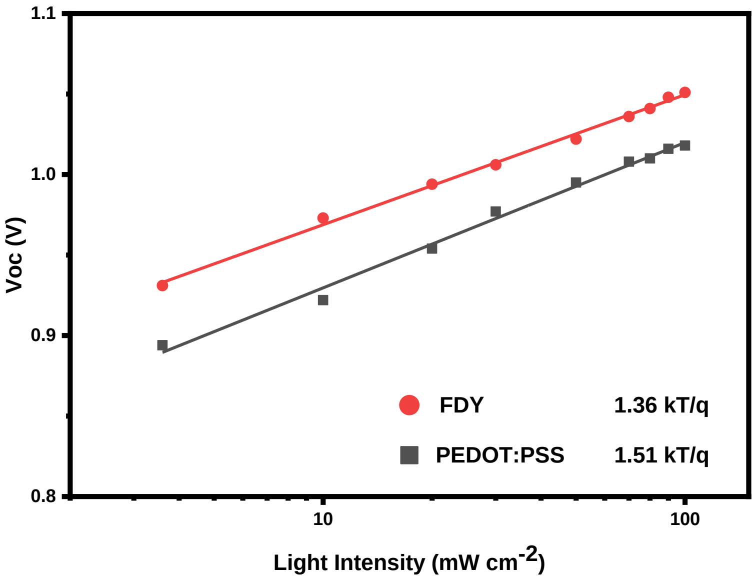

Figure S6 The light intensity dependence of *J–V* characteristics of devices on PETDOT:PSS and FDY as HTMs.

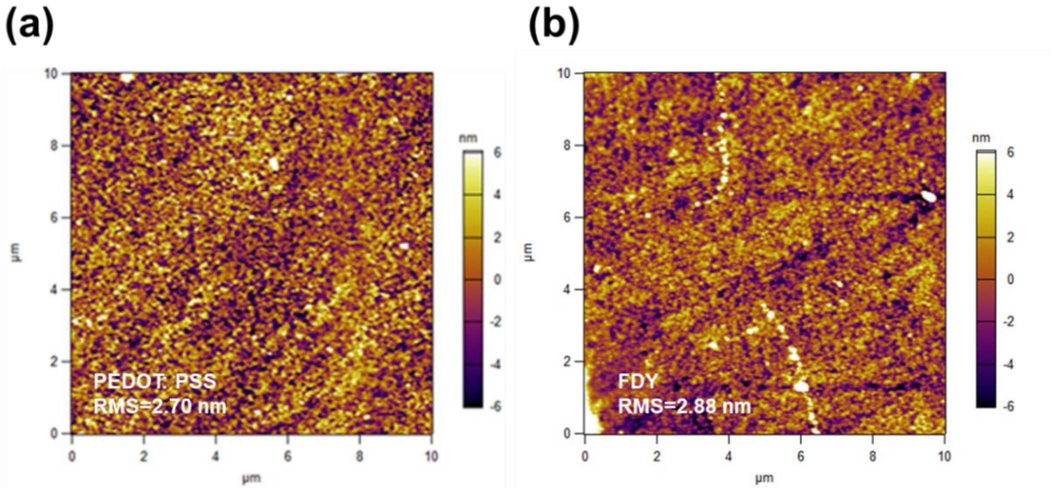

Figure S7. AFM images of the surface morphology of various HTM films fabricated on ITO substrate. a) PEDOT: PSS, b) FDY.

Table S1. Performances of PSCs Based on PEDOT:PSS and FDY at different

concentrations.

HTL	Concentration mg mL^{-1}	J_{sc} [mA cm^{-2}]	V_{oc} [V]	FF [%]	Best PCE [%]	Average PCE[a)][%]
PEDOT:PSS	/	19.38	1.02	78.08	15.41	14.31±0.50
	1	20.07	1.06	81.88	17.42	16.89±0.47
FDY	2	22.42	1.05	79.31	18.69	17.82±0.34
	4	21.21	1.04	77.84	17.17	16.42±0.55

a) The average PCE was obtained from 10 cells

Table S2. TRPL fitting data of perovskite films on PEDOT:PSS and FDY.

samples	A_1	τ_1 (ns)	A_2	τ_2[a)] (ns)	τ_{avg}[b)] (ns)
PVK[c)]	0.46	11.16	0.52	57.65	50.86
PVK/PEDOT:PSS	0.68	8.11	0.34	14.48	11.11
PVK/ FDY	0.30	2.86	0.68	25.28	24.21

a) Average lifetime τaverage= $\Sigma\alpha_i\tau_i$, where α_i= $A_i\tau_i$ /$\Sigma A_i\tau_i$; b) PL quenching efficiency, the fitting formular is $\eta_{quench} = \frac{PL_{bare} - PL_{quench}}{PL_{bare}}$, Where PL_{quench} and PL_{bare} are integrated PL intensities of perovskite on glass substrate with and without HTM, respectively; c) Perovskite.
